# Eine ungewöhnliche Ursache für eine Nierentransplantation

**DOI:** 10.1007/s00108-025-02019-8

**Published:** 2025-11-18

**Authors:** Phillip Kremer, Thorsten Wiech, Ina Kötter, Malte Kluger, Tobias Huber, Martin Krusche, Tingting Xiong

**Affiliations:** 1 III. Medizinische Klinik, Sektion für Rheumatologie und entzündliche Systemerkrankungen, Universitätsklinikum Hamburg-Eppendorf, Martinistraße 52, 20246 Hamburg, Deutschland; 2 Institut für Pathologie, Sektion Nephropathologie, Universitätsklinikum Hamburg-Eppendorf, Hamburg, Deutschland; 3 III. Medizinische Klinik, Nephrologie und Transplantationsmedizin, Universitätsklinikum Hamburg-Eppendorf, Hamburg, Deutschland

**Keywords:** Chronische Nierenerkrankung, Inflammation, Genetik, Seltene Erkrankungen, Autoinflammatorische Erkrankung, Chronic kidney disease, Inflammation, Genetics, Rare diseasen, Autoinflammatory disease

## Abstract

**Hintergrund:**

Systemische Amyloidosen stellen eine heterogene Gruppe seltener Erkrankungen dar, die durch Ablagerungen fehlgefalteter Proteine charakterisiert sind. Die Serum Amyloid A-(AA)-Amyloidose ist Folge chronischer Inflammationsprozesse, wobei das Vorliegen autoinflammatorischer Erkrankungen, insbesondere des familiären Mittelmeerfiebers (FMF), eine der häufigsten Ursachen darstellt. Insbesondere bei Patient:innen aus Gebieten mit hoher Prävalenz und unklarer Niereninsuffizienz sollten genetisch bedingte autoinflammatorische Erkrankungen differenzialdiagnostisch berücksichtigt werden.

**Fallbericht:**

Wir berichten über den Fall einer 50-jährigen Patientin armenischer Herkunft mit Zustand nach Nierentransplantation 2022 bei terminaler Niereninsuffizienz unklarer Genese. Trotz adäquater Immunsuppression zeigte sich eine zunehmende Transplantatdysfunktion mit progredienter Proteinurie. Zusätzlich zeigten sich persistierend erhöhte serologische Entzündungsparameter – allerdings ohne begleitende klinische Symptome. In der wiederholten Transplantatbiopsie gelang letztlich mittels Kongorotfärbung der Nachweis glomerulärer Amyloidablagerungen; immunhistochemisch bestätigte sich eine AA-Amyloidose. Eine genetische Analyse ergab eine Compound-Heterozygotie im MEFV-Gen (Met694Val/Val726Ala). Wir stellten die Diagnose eines familiären Mittelmeerfiebers Typ II mit systemischer Amyloidose und initiierten eine antiinflammatorische Therapie mit Colchicin. Das Typ-II-FMF verläuft primär subklinisch und manifestiert sich erstmals über das Auftreten einer systemischen, meist renalen Typ-AA-Amyloidose.

**Schlussfolgerung:**

Der vorliegende Fall verdeutlicht die Notwendigkeit einer sorgfältigen differenzialdiagnostischen Abklärung bei progredienter Niereninsuffizienz unklarer Genese in Kombination mit persistierender serologischer Inflammation. Bei terminaler Niereninsuffizienz unklarer Ursache und systemischen Entzündungszeichen sollte auch an seltene Ursachen wie eine AA-Amyloidose gedacht werden – insbesondere bei familiärer oder ethnisch bedingter Prädisposition kann eine genetische Diagnostik für FMF sinnvoll sein.

## Abstract (en)

**Background:**

Systemic amyloidoses are a heterogeneous group of rare diseases characterized by deposition of misfolded proteins. Amyloid A (AA) amyloidosis results from chronic inflammatory processes, with autoinflammatory diseases—particularly familial Mediterranean fever (FMF)—representing one of the most common causes. Genetic autoinflammatory diseases should be considered in the differential diagnosis, especially in patients from high-prevalence regions with unexplained renal insufficiency.

**Case report:**

The case of a 50-year-old female patient of Armenian origin who underwent kidney transplantation in 2022 for end-stage renal failure of unknown etiology is reported. Despite adequate immunosuppression, she developed progressive graft dysfunction with increasing proteinuria. In addition, persistently elevated serological inflammatory markers were observed, but without accompanying clinical symptoms. Repeat transplant biopsy ultimately revealed glomerular amyloid deposits on Congo red staining, and immunohistochemistry confirmed AA amyloidosis. Genetic analysis demonstrated compound heterozygosity in the Met694Val/Val726Ala (MEFV) gene. A diagnosis of familial Mediterranean fever type II with systemic amyloidosis was established, and anti-inflammatory therapy with colchicine was initiated. Type II FMF typically follows a primarily subclinical course and first manifests through the development of systemic, most commonly renal, AA amyloidosis.

**Conclusion:**

This case highlights the need for a thorough differential diagnostic work-up in progressive renal failure of unknown etiology combined with persistent serological inflammation. In end-stage renal failure of unknown cause and systemic inflammatory signs, rare conditions such as AA amyloidosis should also be considered. Genetic testing for FMF may be useful, particularly in the presence of familial or ethnic predisposition.

## Fallbericht

Eine 50-jährige Patientin stellte sich Ende 2023 aufgrund von Zephalgien und Übelkeit bei hypertensiver Entgleisung notfallmäßig über unsere zentrale Notaufnahme vor. Laborchemisch zeigten sich eine akut-auf-chronische Nierentransplantatfunktionsverschlechterung (Kreatinin 1,71 mg/dl, Baseline-Kreatinin 1,0 mg/dl) mit mittelgradiger Protein- und Albuminurie (Urin-Albumin-Kreatinin-Ratio 900 mg/g, Urin-Protein-Kreatinin-Ratio 1940 mg/g) und erhöhte Inflammationsparameter (CRP 74 mg/l). Initial hielten wir aufgrund genannter Klinik eine hypertensive Enzephalopathie, eine intrakranielle Blutung sowie einen Infekt unter Immunsuppression für wahrscheinliche Differenzialdiagnosen.

**Abb. 1:**
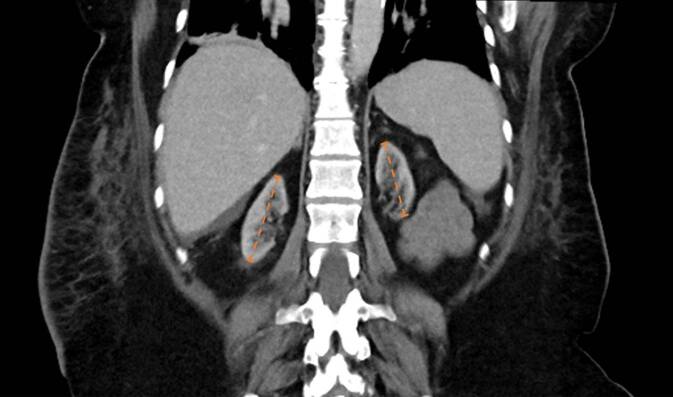
Computertomographie des Abdomens nach erfolgter Nierentransplantation im Oktober 2022. Dargestellt sind bilaterale Schrumpfnieren, rechts 64 mm und links 58 mm im Längsdurchmesser

Die Patientin war im Jahr 2022 bei terminaler Niereninsuffizienz (CKD-Stadium 5, Dialysepflichtigkeit unklarer Genese seit 2014) einer Nierentransplantation unterzogen worden. Aufgrund von bilateralen Schrumpfnieren als Ausdruck einer lange bestehenden Nierenerkrankung im terminalen Zustand konnte eine Nierenbiopsie zur ätiologischen Sicherung nicht durchgeführt werden (siehe Abb. 1). Die aktuelle immunsuppressive Therapie umfasste Prednisolon (5 mg), den Calcineurininhibitor Tacrolimus (5 mg 1‑0-1) sowie den mTOR-Inhibitor Everolimus (3 mg 1‑0-1).

**Abb. 2:**
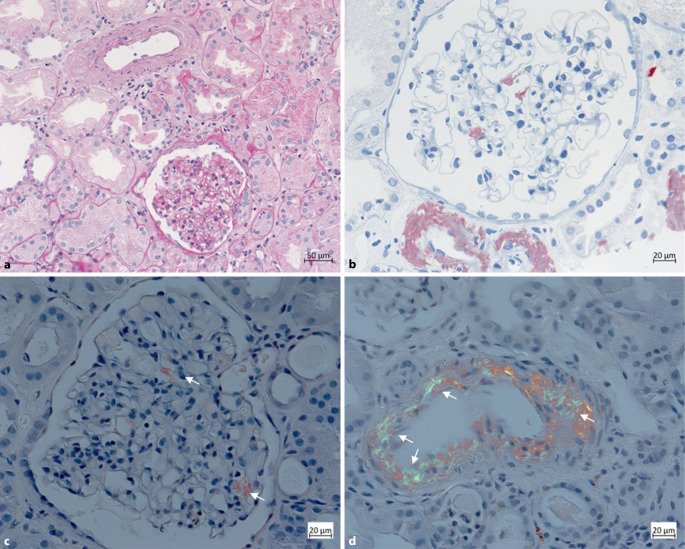
Nierenbiopsie der Transplantatniere: **a** PAS-Färbung, **b** immunhistochemische Färbung mit Nachweis von SAA-Ablagerungen, **c** Kongorotfärbung unter dem Polarisationsmikroskop mit glomerulären Amyloidablagerungen, **d** Kongorotfärbung unter dem Polarisationsmikroskop mit vaskulären Amyloidablagerungen, *Pfeile* markieren diskrete glomeruläre in **c** und mäßiggradige vaskuläre Amyloidablagerungen in **d** mit typischer doppelbrechender, apfelgrüner Farbe

Die Immunsuppressivaspiegel lagen zum Zeitpunkt der aktuellen Vorstellung im Zielbereich (Tacrolimus: 5,7 µg/l, Ziel 5–7 µg/l; Everolimus: 4,3 µg/l, Ziel 4–6 µg/l). Wir stellten die Indikation zur Biopsie der Transplantatniere, in welcher sich histologisch eine mittelgradige zelluläre Rejektion mit Infiltraten an der Markrindengrenze und eine mittelgradige interstitielle Fibrose mit Tubulusatrophie (IFTA) zeigten. Eine Intensivierung der immunsuppressiven Therapie mittels Prednisolonstoß und Steigerung der Immunsuppressiva folgte. Im November 2024 erfolgte eine erneute Hospitalisierung der Patientin. Aufgrund einer anhaltenden Nierenfunktionseinschränkung (Kreatinin 2,03 mg/dl) stellten wir die Indikation zur Rebiopsie. In der histopathologischen Untersuchung der Rebiopsie kamen in der Kongorotfärbung glomeruläre und vaskuläre Ablagerungen von Amyloid mit typischem „apfelgrünem“ Erscheinungsbild in der Polarisationsmikroskopie zur Darstellung (siehe Abb. 2c,d). In der Immunhistochemie gelang der Nachweis von Amyloid A (siehe Abb. 2b).

**Abb. 3:**
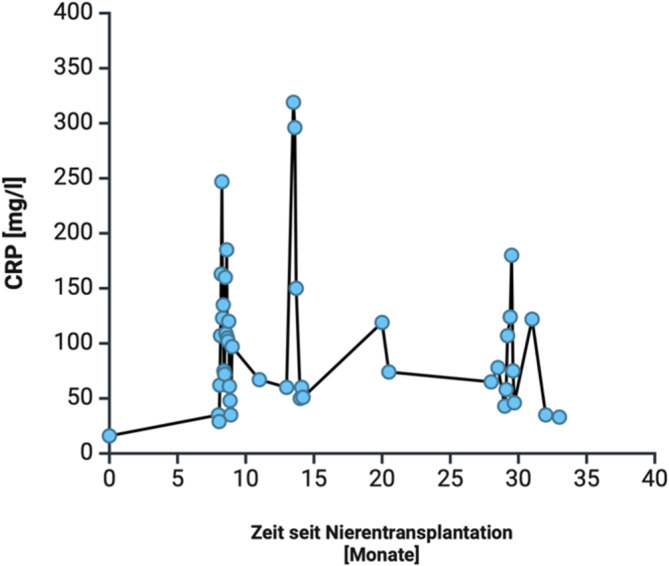
CRP-Verlauf seit Nierentransplantation im Oktober 2022 (Zeitpunkt 0)

Nach retrospektiver Beurteilung des Krankheitsverlaufs fiel auf, dass die Patientin seit Erstvorstellung in unserer Klinik (Februar 2022, Transplantation Oktober 2022) zu keinem Zeitpunkt normwertige serologische inflammatorische Marker (CRP und Leukozyten) aufwies (siehe Abb. 3). Eindrucksvollerweise berichtete die Patientin, subjektiv vollkommen beschwerdefrei zu sein. In der Familienanamnese gab es keine Hinweise auf autoimmunologische oder autoinflammatorische Erkrankungen. In der Vergangenheit war die Patientin einer Appendektomie unterzogen worden. Der darüber hinaus bestimmte Serum-Amyloid-A-Wert lag deutlich oberhalb des oberen Referenzbereichs (SAA 126 mg/l, Referenzbereich < 8 mg/l).

**Abb. 4:**
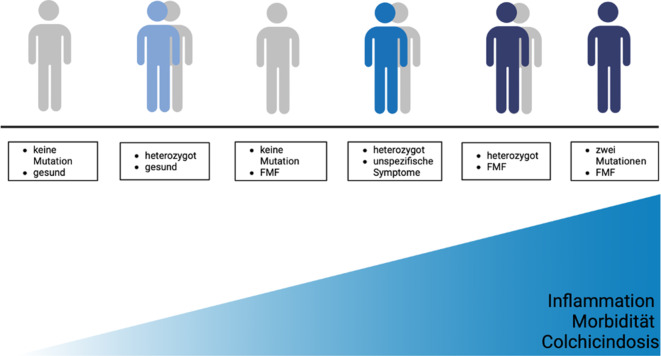
Genotyp-Phänotyp-Korrelation beim familiären Mittelmeerfieber, adaptiert nach Kallinich et al. [25]

Wir stellten die Diagnose einer systemischen Amyloidose vom Typ Serum-Amyloid A (AA-Amyloidose). Ein intravenöser Drogenabusus oder rezidivierende Infektionen lagen nicht vor.

Aufgrund des ethnischen Hintergrunds der Patientin (Geburtsland Armenien, 2014 Immigration) und der dortigen erhöhten Prävalenz des familiären Mittelmeerfiebers erfolgte eine genetische Untersuchung auf pathogene Varianten im MEFV-Gen. Hier zeigte sich eine Compound-Heterozygotie mit Nachweis der Varianten *Met694Val* und *Val726Ala* (Einstufung als hochpathogen anhand der ACMG/AMP-Kriterien und [1]). In Zusammenschau der Befunde stellten wir die Diagnose eines familiären Mittelmeerfiebers vom Typ II mit systemischer Amyloidose vom Typ AA. Eine antiinflammatorische Therapie mit Colchicin (0,5 mg 1‑0-1) wurde eingeleitet. Hierunter kam es zu einem deutlichen Abfall des SAA auf 18,3 mg/l (Referenzbereich < 8 mg/l). In der weiterführenden Diagnostik fiel echokardiographisch eine konzentrische, linksventrikuläre (LV) Hypertrophie (interventrikuläres Septum diastolisch [IVSd] 27,0 mm, LV-Masse 290 g) mit höhergradiger diastolischer Dysfunktion (E/A 0,77) auf. In der kardialen MRT-Bildgebung konnte bei ischämischem Late-Gadolinium-Enhancement (LGE) inferolateral eine myokardiale Beteiligung nicht bestätigt werden. Wir werteten die LV-Hypertrophie demnach im Rahmen der langjährigen, unkontrollierten arteriellen Hypertonie und Dialysepflichtigkeit. Hinweise auf weitere Organbeteiligungen (GI-Trakt, Milz, Polyneuropathie) ergaben sich anhand des klinischen Bilds und bildgebender Befunde nicht.

## Diskussion

### AA-Amyloidose

Amyloidosen stellen eine heterogene Gruppe von seltenen Erkrankungen dar, bei denen es zur lokalen oder systemischen Ablagerung von fehlgefalteten Proteinen und nachfolgender teils irreversibler Organschädigung kommt [2]. Zum heutigen Zeitpunkt sind 42 amyloidogene Proteine beschrieben [3]. Aufseiten der systemischen Amyloidosen stellen die Leichtkettenamyloidose (AL-Amyloidose), die Serum-Amyloid-A-Amyloidose (AA-Amyloidose), die Transthyretin-Amyloidose (ATTR-Amyloidose, Wildtyp [ATTRwt] oder hereditär [ATTRv]) und die β2-Mikroglobulin(β2M)-Dialyse-assoziierte Amyloidose die relevantesten Erkrankungsbilder dar.

AA-Amyloidosen, die etwa 12 % der systemischen Amyloidosen ausmachen, entstehen überwiegend infolge chronisch-entzündlicher Erkrankungen und chronischer Infektionen, die mit einer langanhaltenden starken Erhöhung des Akute-Phase-Proteins Serum-Amyloid A (SAA) einhergehen [2]. Zu den häufigsten assoziierten Erkrankungen zählen chronisch-entzündliche rheumatische Erkrankungen (z. B. rheumatoide Arthritis), chronisch-entzündliche Darmerkrankungen sowie autoinflammatorische Syndrome wie das familiäre Mittelmeerfieber (siehe Tab. 1; [4]). In Regionen mit hoher Infektionsprävalenz sind chronische Infektionen wie Tuberkulose, Osteomyelitis und chronische Hautinfektionen relevante Ursachen. Eine unterschiedliche Häufigkeit der jeweiligen Ursache sollte je nach geografischer Lage berücksichtigt werden.

**Tab. 1:** Ursachen der AA-Amyloidose.

Ursache	Beispiele Erkrankung	Häufigkeit (in %)	Referenz
Autoimmune, rheumatologische Systemerkrankungen	Rheumatoide Arthritis, juvenile idiopathische Arthritis, axiale Spondylarthritis, Psoriasisarthritis	30–40	[24, 31–33]
Chronische Infektionen	Bronchiektase, Tuberkulose, Osteomyelitis, chronische Harnwegsinfektionen, Lepra, HIV	5–20	[31, 32, 34, 35]
Intravenöser Drogenabusus	–	10	[33, 36]
Monogenetische autoinflammatorische Erkrankungen	Familiäres Mittelmeerfieber, TNF-Rezeptor-assoziiertes periodisches Syndrom (TRAPS), cryopyrinassoziiertes periodisches Syndrom (CAPS)	5–25	[24, 31, 32, 34]
Maligne Erkrankungen	Lymphom, Nierenzellkarzinom, Adenokarzinom der Lunge, Niere, Magen-Darm-Trakt	2–7	[31, 34, 37]
Chronisch-entzündliche Darmerkrankungen	Morbus Crohn, Colitis ulcerosa	2–7	[31, 32, 34]
Idiopathisch	Ursache nicht kausal identifiziert	10–15	[24, 31, 32, 34]

Pathophysiologisch kommt es unter dem Einfluss proinflammatorischer Zytokine (insbesondere IL‑1, IL‑6, TNF-α) zu einer vermehrten Produktion von SAA in der Leber [5]. Persistierend erhöhte SAA-Konzentrationen im Blutkreislauf führen im Tiermodell zu einer beschleunigten Entstehung einer AA-Amyloidose. Dabei korrelieren höhere SAA-Spiegel mit einer schnelleren Krankheitsprogression [6]. Nur ein Teil der Patient:innen mit chronisch erhöhtem SAA entwickelt eine Amyloidose. Risikofaktoren hierfür sind die Dauer und Höhe der SAA-Erhöhung sowie genetische Polymorphismen im Gen *SAA1* [7, 8]. In bis zu 20 % der Fälle bleibt die Ursache der AA-Amyloidose unklar (idiopathisch), wobei genetische Faktoren eine Rolle spielen können [9].

Klinisch kommt es bei über 90 % der Patient:innen mit AA-Amyloidose zu einer renalen Beteiligung [5]. In der frühen Krankheitsphase liegt häufig lediglich eine milde Proteinurie (Protein-Kreatinin-Ratio 100–300 mg/g) vor. Bei Fortschreiten der Erkrankung kann es zunächst zu einer moderaten Proteinurie (Protein-Kreatinin-Ratio 300–1000 mg/g) und schließlich zur typischen Manifestation des nephrotischen Syndroms (Protein-Kreatinin-Ratio > 3500 mg/g) sowie zu einer terminalen Niereninsuffizienz kommen. Auch die in 70 % der Fälle bestehende gastrointestinale Beteiligung zeigt sich häufig initial asymptomatisch, wobei im fortgeschrittenen Verlauf Malabsorptionssyndrome, chronische Diarrhöen und progrediente Kachexie auftreten können [5]. Eine kardiale Beteiligung im Rahmen der AA-Amyloidose ist im Vergleich zur AL- oder ATTR-Amyloidose eher seltener und liegt in etwa 10 % der Fälle vor [5]. Die Diagnose kann durch den histopathologischen Nachweis von Amyloid in der Kongorotfärbung und Amyloidfibrillen in der Elektronenmikroskopie gestellt werden [5]. Besonders geeignete Lokalisationen für eine Biopsie sind hierbei Bauchfett, Niere, Duodenum und Rektum. Die empfohlene Therapie der AA-Amyloidose besteht primär in der konsequenten Kontrolle und Behandlung der zugrunde liegenden chronisch-entzündlichen Erkrankung, um die Produktion des Serum-Amyloid-A-Proteins (SAA) zu senken.

### Familiäres Mittelmeerfieber

Das familiäre Mittelmeerfieber (FMF) ist eine autoinflammatorische Erkrankung, die auf pathogenen Mutationen im *Mediterranean-fever*-Gen (*MEFV*; kodierendes Protein „Pyrin“) beruht. Typische klinische Manifestationen sind Bauchschmerzen (Peritonitis), Brustschmerzen (Pleuritis, seltener Perikarditis), Arthritis und gelegentlich erysipelartige Hautveränderungen. Die Attacken dauern in der Regel 12–72 h und treten meist im Kindes- oder Jugendalter erstmals auf, können aber auch im Erwachsenenalter auftreten [10]. Zwischen den Schüben sind die Betroffenen in der Regel beschwerdefrei. Im Rahmen der Schübe zeigt sich typischerweise eine Erhöhung der Inflammationsparameter (C-reaktives Protein [CRP], SAA, IL-18), wobei in Einzelfällen auch bei asymptomatischen Patient:innen eine serologische Inflammation nachweisbar sein kann (Abb. 2; [11]). Historisch betrachtet wurde lange Zeit von einem klassischen autosomal-rezessiven Vererbungsmechanismus ausgegangen [12]. Mittlerweile geht man jedoch davon aus, dass eine Genotyp-Phänotyp-Korrelation bzw. Gendosis-Wirkungs-Beziehung mit einem Krankheitskontinuum besteht, wobei die Erkrankung auch bei Vorliegen einer einzelnen pathogenen Variante manifest werden kann [13–15]. Die vier bisher beschriebenen und validiert hochpathogenen Varianten sind M694V, M680I, M694I und V726A [16]. Die Heterozygotenfrequenz für *MEFV-*Mutationen ist in einigen Ländern des Mittelmeerraums (darunter Israel, Türkei, Armenien) am höchsten und liegt bei 1:5 bis 1:7 [14, 17].

Auf Grundlage klinischer und genetischer Merkmale können zum aktuellen Zeitpunkt drei verschiedene FMF-Typen (Typ I, II und III) differenziert werden [12, 18]. Das Typ-I-FMF kennzeichnet sich durch FMF-typische Beschwerden (Fieber und serositisassoziierte Beschwerden), das Typ-II-FMF verläuft primär subklinisch und manifestiert sich erstmals über das Auftreten einer systemischen, meist renalen, Typ-AA-Amyloidose [12]. Patient:innen mit Typ-II-FMF haben in aller Regel keine typischen Beschwerden (s. oben) und sind meist beschwerdefrei. Laborchemisch fallen erhöhte Inflammationsparameter auf. Eine infektiologische Abklärung bleibt in der Regel erfolglos. Im Verlauf der Krankheitshistorie entwickeln Patient:innen nach vielen Jahren eine AA-Amyloidose, welche sich initial über eine Proteinurie oder Nierenfunktionsverschlechterung bemerkbar macht. Das typische Alter, in dem Patient:innen mit Typ-II-FMF eine terminale Niereninsuffizienz mit Dialysepflichtigkeit entwickeln, liegt meist zwischen dem 20. und 40. Lebensjahr. Die Progression zur Dialysepflichtigkeit hängt stark von genetischen Faktoren (insbesondere *M694V*-Mutationen) sowie dem Zeitpunkt der Diagnosestellung und Therapiecompliance ab [19]. Ein Typ-III-FMF liegt bei Erwachsenen vor, bei denen trotz Nachweis von zwei hochpathogenen Mutationen (homo- oder heterozygot) weder klinische FMF-Beschwerden noch eine Amyloidose vorliegen [18]. Die Diagnosestellung erfolgt anhand der Eurofever-PRINTO-Kriterien (siehe Tab. 2; [20]).

**Tab. 2:** Eurofever-PRINTO-Kriterien [20].

Vorhandensein eines bestätigten MEFV-Genotyps* und mindestens einer der folgenden Punkte:
Dauer der Episoden 1–3 Tage
Arthritis
Thorakale Schmerzen
Abdominelle Schmerzen
Vorhandensein eines *nicht bestätigten MEFV-Genotyps*^*‡*^ und mindestens zwei der folgenden Punkte:
Dauer der Episoden 1–3 Tage
Arthritis
Thorakale Schmerzen
Abdominelle Schmerzen
**Sensitivität:** 0,94 **Spezifität:** 0,95 **„Accuracy“:** 0,98

*Nachweis einer „pathogenic“ Variante oder „likely pathogenic“-Variante.

^‡^In trans compound-heterozygot für eine pathogene MEFV-Variante und eine Variante unklarer Signifikanz (VUS) oder biallelisch VUS oder heterozygot für eine pathogene MEFV-Variante.

Die Entstehung einer AA-Amyloidose stellt die schwerste Langzeitkomplikation des FMF dar und verdeutlicht die enge Assoziation der beiden Erkrankungen [21, 22]. Das Risiko, eine AA-Amyloidose zu entwickeln, wird in erster Linie durch genetische Faktoren bestimmt, insbesondere durch die jeweilige Variante im MEFV-Gen [12, 23]. Dabei ist die pathogene Variante M694V, insbesondere im homozygoten Zustand, der wichtigste genetische Risikofaktor, der im Vergleich zu anderen Genotypen ein deutlich erhöhtes Amyloidoserisiko mit sich bringt [23]. Ein zusätzliches Risiko geht von bestimmten SAA1-Genotypen aus, insbesondere SAA1.1/1.1, was die Auftretenswahrscheinlichkeit für eine Amyloidose bei FMF-Patienten weiter erhöht [24, 25]. In einer prospektiven Langzeitstudie von Lachmann et al., in der 374 Patient:innen mit AA-Amyloidose untersucht wurden, korrelierte die Höhe der SAA-Spiegel signifikant mit der renalen Prognose, dem „amyloid burden“ und der Mortalität [26]. Bei 60 % der Patient:innen mit normwertigen SAA-Werten zeigte sich eine Regredienz der Amyloidablagerungen im Verlauf [26].

In der Therapie des FMF ist Colchicin der Grundbaustein in der Anfallsprävention und der Amyloidoseprophylaxe [27]. Eine lebenslange tägliche Colchicintherapie (in der Regel 1,0–3,0 mg/Tag bei Erwachsenen, mit Dosisanpassung bei Nieren- oder Leberfunktionsstörungen) ist meist wirksam in der Verhinderung von Amyloidablagerungen und des weiteren Fortschreitens bei Vorliegen einer chronischen Niereninsuffizienz, insbesondere bei Hochrisikogenotypen wie M694V-Homozygoten [26]. Eine Colchicinresistenz^1^ oder die Nichteinhaltung der Therapie erhöht das Risiko einer Amyloidose deutlich. In colchicinresistenten Fällen sollte eine zusätzliche Interleukin-1(IL-1)-Blockade mittels Anakinra oder Canakinumab erfolgen [27, 28]. Im Falle von anhaltend erhöhten SAA-Werten sollte ebenso die Compliance der Medikamenteneinnahme kritisch geprüft werden.

Eine engmaschige Überwachung der Proteinurie und eine regelmäßige Beurteilung der Nierenfunktionsparameter sind bei FMF-Patient:innen unerlässlich, insbesondere bei Hochrisikogenotypen oder insuffizientem Ansprechen auf Colchicin. Eine klinische Schubfreiheit sowie vollständige Normalisierung der serologischen Inflammationsparameter (CRP, SAA) sollte angestrebt werden [27]. Bei Auftreten einer renalen AA-Amyloidose mit terminaler Niereninsuffizienz sollte eine Nierentransplantation erwogen werden, sofern die Grunderkrankung gut eingestellt ist. Eine retrospektive Analyse von 86 Patient:innen, die aufgrund einer AA-Amyloidose nierentransplantiert wurden (hiervon 43 % mit FMF), zeigt insgesamt ein sehr gutes Gesamt- sowie Transplantatüberleben mit einer histologisch gesicherten Rekurrenz der AA-Amyloidose in 5,8 % der Gesamtkohorte [29]. Eine weitere monozentrische retrospektive Analyse von 17 Patient:innen nach Nierentransplantation bei FMF mit AA-Amyloidose ergab ein 89 %iges Graft- und 90 %iges Patient:innenüberleben nach 5 Jahren unter Colchicintherapie [30]. Unter konsequenter antiinflammatorischer Therapie ergibt sich bei Patient:innen somit eine geringe Rekurrenz im Nierentransplantat und ein ausgezeichnetes Langzeitüberleben.

Zusammenfassend sollte bei Patient:innen mit unklarer chronischer Nierenerkrankung und (sub-)klinischer Inflammation differenzialdiagnostisch eine AA-Amyloidose in Betracht gezogen werden. Eine entsprechende genetische Panel-Diagnostik kann bei unklarer chronischer Nierenerkrankung initiiert und über die gesetzliche Krankenkasse abgerechnet werden. Das familiäre Mittelmeerfieber ist bei entsprechendem ethnischem Hintergrund eine häufige Ursache.

## Fazit für die klinische Praxis

- *AA-Amyloidose als Differenzialdiagnose bei unklarer CKD: *Bei Patient:innen mit chronischer Niereninsuffizienz unklarer Genese und persistierenden Entzündungszeichen sollte frühzeitig auch an eine AA-Amyloidose gedacht werden.
- *Familiäres Mittelmeerfieber (FMF) kann sich primär über eine Amyloidose manifestieren (Typ II):* Ein FMF sollte auch bei zunächst fehlender typischer Klinik als Ursache einer systemischen AA-Amyloidose in Erwägung gezogen werden. Typ II manifestiert sich ausschließlich über die Amyloidose – die genetische Diagnostik ist essenziell.
- *SAA als zentraler Verlaufsparameter: *Dauerhaft erhöhte Serum-Amyloid-A-Werte sind ein sensitiver Marker für das Risiko einer Amyloidose und sollten routinemäßig bei Verdacht auf autoinflammatorische Erkrankungen bestimmt und kontrolliert werden.
- *Histologie bleibt Goldstandard zur Diagnosesicherung:* Trotz moderner Bildgebung und Serologie ist der histologische Kongorotnachweis mit immunhistochemischer Typisierung (z. B. AA) der entscheidende Schritt zur Diagnosesicherung bei Amyloidoseverdacht – auch im Rahmen einer Rebiopsie nach Nierentransplantation.

## Footnotes

1 *Definition der Colchicinresistenz nach EULAR*: Eine Colchicinresistenz liegt vor, wenn unter adäquater Colchicindosis und guter Adhärenz weiterhin durchschnittlich ≥ 1 Schub/Monat über mindestens drei Monate oder persistierende Entzündungszeichen bestehen.
